# Metagenomic Characterization of Intestinal Regions in Pigs With Contrasting Feed Efficiency

**DOI:** 10.3389/fmicb.2020.00032

**Published:** 2020-01-23

**Authors:** Jianping Quan, Zhenfang Wu, Yong Ye, Longlong Peng, Jie Wu, Donglin Ruan, Yibin Qiu, Rongrong Ding, Xingwang Wang, Enqin Zheng, Gengyuan Cai, Wen Huang, Jie Yang

**Affiliations:** ^1^College of Animal Science and National Engineering Research Center for Breeding Swine Industry, South China Agricultural University, Guangzhou, China; ^2^Department of Animal Science, College of Agriculture and Natural Resources, Michigan State University, East Lansing, MI, United States

**Keywords:** feed efficiency, intestinal microbiome, metagenome, *macB* gene, DLY pigs

## Abstract

Greater feed efficiency (FE) is critical in increasing profitability while reducing the environmental impact of pig production. Previous studies that identified swine FE-associated bacterial taxa were limited in either sampling sites or sequencing methods. This study characterized the microbiomes within the intestine of FE contrasting Duroc × (Landrace × Yorkshire) (DLY) pigs with a comprehensive representation of diverse sampling sites (ileum, cecum, and colon) and a metagenomic sequencing approach. A total of 226 pigs were ranked according to their FE between weaning to 140 day old, and six with extreme phenotypes were selected, three for each of the high and low groups. The results revealed that the cecum and colon had similar microbial taxonomic composition and function, and had higher capacity in polysaccharide metabolism than the ileum. We found in cecum that the high FE pigs had slightly higher richness and evenness in their micriobiota than the low FE pigs. We identified 12 phyla, 17 genera, and 39 species (e.g., *Treponema porcinum*, *Treponema bryantii*, and *Firmicutes bacterium CAG:110*) that were potentially associated with swine FE variation in cecum microbiota through LEfSe analysis. Species enriched in the cecum of the high FE pigs had a greater ability to utilize dietary polysaccharides and dietary protein according to the KEGG annotation. Analysis of antibiotic resistance based on the CARD database annotation indicated that the *macB* resistant gene might play an important role in shaping the microbial community in the cecum of pigs with contrasting FE. The bacteria from the genus *Prevotella* was highly enriched in the cecum of low FE pigs, which may impair the establishment of a more effective nutrient harvesting microbiota because of the interaction between *Prevotella* and other benefical microbes. These findings improved our understanding of the microbial compositions in the different gut locations of DLY pigs and identified many biomarkers associated with FE variation wich may be used to develop strategies to improve FE in pigs.

## Introduction

The pig is an important food animal, which provides ∼36% of all meat utilization by the world population in 2018 (FAO, 2019). In pig production, the feed accounts for ∼70% of the total cost (Teagasc, 2018). Greater feed efficiency (FE) would increase profitability while reducing the environmental impact of pig production. This is especially important given that pig is one of the major sources of meat in human diet.

Feed efficiency is a complex phenotype influenced by genetic and environmental factors, including nutrition, management, and the physiological and health status of the animals (Armstrong et al., 2000; Martinez et al., 2009; Oliveira et al., 2009; Ding et al., 2018). Thus, strategies to improve FE may target one or more of these factors.

The involvement of gut microbiota in host metabolism and health is well accepted (Lynch and Pedersen, 2016), but the specific roles remain to be defined. For example, germ-free mice transplanted with uncultured fecal samples or corresponding cultured bacterial collection from obese mice had increased body and fat mass and developed obesity-related metabolic disorders, compared to the germ-free mice transplanted with uncultured fecal samples from lean mice (Ridaura et al., 2013), indicating potentially a critical role of microbiome in modulating metabolism. Furthermore, the rumen of ruminants harbors micro-organisms that possess the necessary enzymes to digest cellulose in their diets (Wang et al., 2019). Finally, multiple previous studies in pigs including ours found that the gut microbiome of pigs with higher FE contained more short chain fatty acid (SCFA)-producing bacteria (Tan et al., 2017; Yang et al., 2017; Quan et al., 2018), suggesting an important role of enzymatic activities of the microbiome in regulating FE. However, the exact mechanisms remain to be defined. Because of the large contribution of SCFA by cellulytic bacteria (Dierick et al., 1989), these particular bacteria may increase the effiency of feed utilization of the hosts. Specifically for FE, the gut microbiome has a clear role in regulating weight gain in humans (Ley et al., 2006).

However, previous studies were limited in either sampling sites or sequencing methods, mostly using fecal samples and 16S rRNA gene sequencing (McCormack et al., 2019; Quan et al., 2019). To better understand the contribution of microbiome to efficient feed conversion, it is necessary to have a comprehensive representation of diverse sampling sites and a sequencing method that better resolves bacteria classification. In this study, we performed metagenomic shotgun sequencing of samples from three intestinal regions (ileum, cecum, and colon) of three-way hybrid Duroc × (Landrace × Yorkshire) (DLY) pigs that exhibited high and low FE. This allowed us to identify microbial and functional differences associated with FE variation in pigs. Because of the popular utilization of DLY pigs in commercial pork production, this study will have strong applied implications.

## Materials and Methods

### Laboratory Animal Management and Sample Collection

The experimental procedures used in this study were approved by the Animal Care and Use Committee (ACUC) of South China Agricultural University (SCAU) (approval number SCAU#0017). FE phenotypes were recorded as previously described (Quan et al., 2018). Briefly, a total of 226 DLY female piglets, which had similar genetic backgrounds were uniformly nursed before they were selected for FE phenotying after normal weaning. Animals were randomly assigned to 30 pens (6–8 per pen) at a fattening house, raised under the same commercial formula diet, and under controlled farm conditions and management. The Osborne’s FIRE (Feed Intake Recording Equipment) System (Osborne Industries Inc., Osborne, Kansas) were used to record daily feed intake and daily body weight gain of each pig. In this study, we defined FE as the weight gained between weaning and 140 days of age, divided by total feed intake, which is the inverse of the commonly used feed conversion ratio (FCR). We selected the top 12 and bottom 12 pigs on the phenotypic distribution in a previous study using 16S rRNA gene sequencing (Quan et al., 2018). These pigs were slaughtered at 141–142 days of age. The content at three gut locations (ileum, cecum, and colon) was collected and the metagenomic DNA was extracted (Quan et al., 2018). We selected three representative high FE and three representative low FE pigs from these two groups, respectively, to maximize their metagenomic diversity, based on their 16S rRNA gene sequencing results and subjected the metagenomic DNA to shotgun DNA sequencing.

### Metagenomic Sequencing and Gene Identification

DNA was extracted using a Soil Genome^TM^ DNA Isolation kit (Qiagen, Germany) following the manufacturer’s instructions. The UV–Vis spectrophotometry (NanoDrop 2000, United States) and the 1% agarose gel electrophoresis were used to measure or evaluate the DNA concentration and integrity. These 18 (3 animals × 3 sites × 2 groups) metagenomic DNA samples were sequenced using 150 bp paired-end sequencing on the Illumina platform. Bioinformatic analyses to identify genes are as follows (Supplementary Figure S1). Sequence reads were first screened to remove low quality reads by Readfq v8^1^ and host genome contamination was removed by mapping reads to the pig reference genome (Sscrofa11.1) using bowtie2 (v2.3.3) (Langmead and Salzberg, 2012), with the parameters –end-to-end –sensitive -I 200 -X 400. The resulting clean data from each of the 18 samples were assembled using SOAPdenovo (v2.04) (Luo et al., 2012) to obtain scaftigs with the parameters -d 1 -M 3 -R -u -F -K 55. All clean data from each sample were aligned by bowtie2 to the scaftigs and the unmapped reads were collected to undergo another SOAPdenovo assembly with the same parameters. The scaftigs (>500 bp) from all single-sample assemblies and the mixed-sample assembly were used to predict genes using MetaGeneMark (v3.38) (Zhu et al., 2010), the parameters were as follow: gmhmmp -a -d -f 3 –m –A -D. The predicted ORFs (>100 nt) were clustered and redundancy was eliminated using CD-HIT (v4.8.1) (Li and Godzik, 2006), the parameter options were -c 0.95 -G 0 -M 0 -T 0 -aS 0.9 -g 1 -d 0. To increase the coverage of microbial genes, we combined the gene catalog from this study with a reference pig gut microbiome gene catalog (Xiao et al., 2016) and removed redundancy using CD-HIT to generate a combined gene catalog. Finally, reads from each sample were mapped again using bowtie2 to this mixed gene catalog and only genes that contained at least one mapped read from any of the 18 samples were retained for subsequent analyses. The retained gene list was defined as the final non-redundant gene catalog in this study.

### Taxonomic Classification and Functional Annotation

The sequences of taxonomy annotation with an bacteria, fungi, archaea, and viruses were extracted from the pre-formatted BLAST NR database (accessed 6 January 2019). Diamond (v0.9.24) (Buchfink et al., 2015) was used to blast sequences of genes included in final gene catalog against the microbiome database to obtain the taxonomy ID profile with the parameters of blastx -e 0.00001 -b 8 -f 102 –top 2. Subsequently, the taxonomy hierarchy (kingdom, phylum, class, order, family, genus, species) was acquired by the taxonomizr R package (Sherrill-Mix, 2019). Functional annotations were performed using Diamond by blasting the gene sequences against functional databases, including the carbohydrate-active enzymes (CAZy) database (v07312018), the comprehensive antibiotic resistance database (CARD) (v12012017) with the parameters of blast -e 0.00001 -b 4. Annotations by the Kyoto Encyclopedia of Genes and Genomes (KEGG) database (v07132018) were performed using the MAPLE online server^2^.

### Quantification of Taxonomic and Functional Abundance

The abundances of genes in final gene catalog were estimated using uniquely mapped fragment counts obtained by bowtie2. The transcripts per million (TPM) (Wagner et al., 2012) for each gene in each sample was calculated as $a_{j}=\frac{b_{j}/c_{j}}{\sum_{j=1}^{n}b_{j}/c_{j}}\times 10^{6}$, where *a*_*j*_ is the TPM of gene *j*; *b*_*j*_ is the number of uniquely mapped fragments in a sample; *c*_*j*_ is the length of gene j; and *n* is the total number of genes. The abundance of each taxonomic hierarchy in one sample is equal to the sum of the gene abundances annotated to it. Similarly, the abundance of each CAZyme, resistance gene, or KEGG orthology (KO) was also calculated as the total abundance of genes annotated to each term.

### Statistical Analysis and Visualization

The alpha diversity indexes are used to estimate the complexity of species diversity in samples (Li et al., 2013). The Chao1 and Shannon indexes calculated using functions in the R package vegan (Oksanen et al., 2019) were used to measure the richness and evenness of species in samples. The Bray–Curtis distance matrixes were calculated using vegan to measure the differences in the microbiome composition between samples from different intestinal sites and FE groups, and the results were visualized by non-metric multidimensional scaling (NMDS) using R (v3.5.0). The analysis of similarities (ANOSIM) also were performed through the vegan package to test whether there is a significant difference between groups. Linear discriminant analysis (LDA) coupled with the effect size (LEfSe) algorithm was used to identify biomarkers that were characteristic of each group based on the abundance values (Segata et al., 2011). *Z*-scores were calculated to construct a heatmap though the R package pheatmap (Kolde, 2018), to demonstrate the abundance of the taxonomic or functional profiles in each group with the formula *z* = (*x* − μ)/σ, where *x* is the abundance of the taxonomic or functional profiles in each group, μ is the mean value of the abundances in all groups, and σ is the standard deviation of the abundances. The Wilcoxon rank sum test was used to determine the significance of the alpha diversity, and the abundance of taxonomy and functional items between the sample groups. The Benjamini–Hochberg FDR method was used to correct the multiple comparisons (Benjamini and Hochberg, 1995). The data visualization in this study was mostly conducted in R environment using ggplot2 (Wickham, 2009).

### Data Availability

The whole genome shotgut sequencing data were deposited in the NCBI’s Sequence Read Archive (SRA) database under the accession ID PRJNA575543.

## Results

### Comparative Metagenomic Sequencing of Pigs With High and Low Feed Efficiency

**FIGURE 1:**
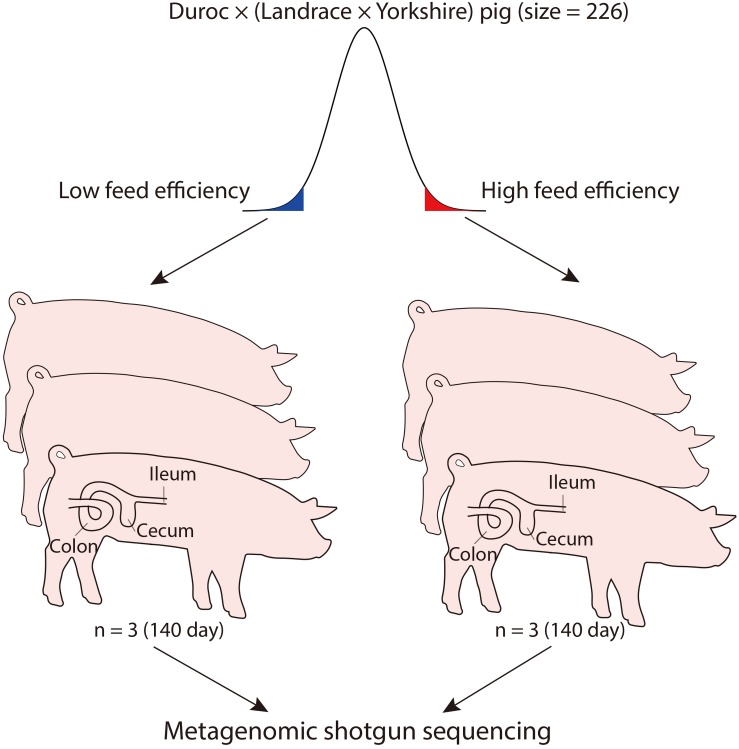
Overview of the experimental design. Feed efficiency (FE) was defined as the weight gained between weaning and 140 days of age, divided by total feed intake.

**FIGURE 2:**
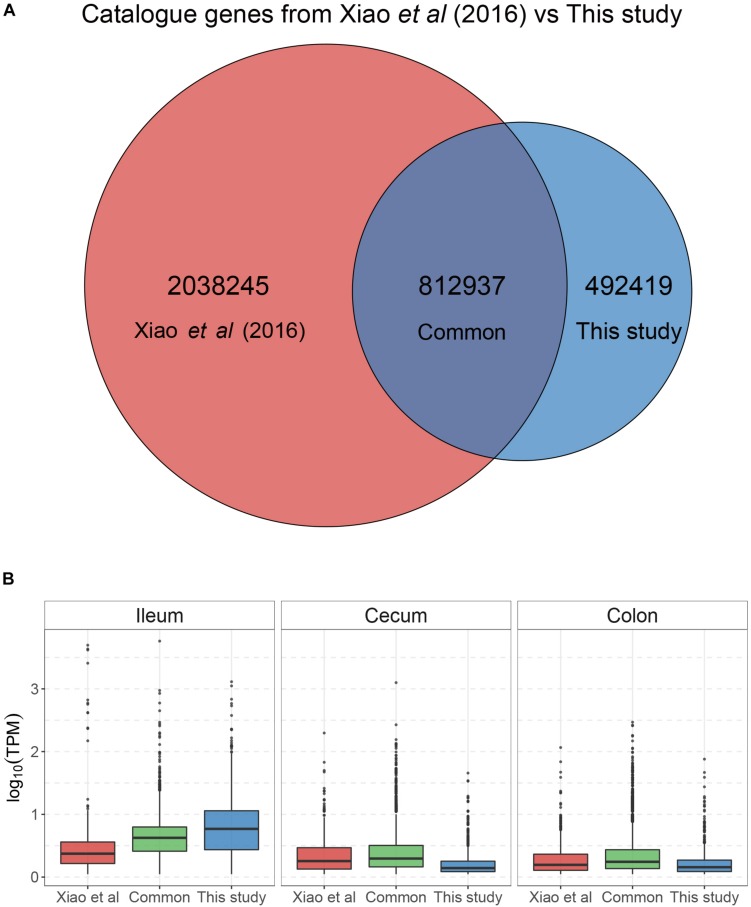
Distribution of abundance of genes in the gene catalog. The gene catalog was constructed by merging genes identified in the present study and a previous reference set (Xiao et al., 2016). **(A)** Venn diagram of intersection between the genes in the reference set and identified in the present study. **(B)** Boxplot showing the distribution of gene abundance in each category based on the Venn diagram.

To identify microbial species and/or functions that are associated with FE variation in pigs, we selected three sows with high FE and three with low FE from a population of 226 DLY pigs (Figure 1 and Supplementary Table S1). Digesta from the ileum, cecum, and colon were sampled from each pig. The metagenomic DNA from these samples were sequenced to an average depth of 68 M 150 bp reads (Supplementary Table S2). The sequence reads from each individual samples were *de novo* assembled to obtain scaftigs >500 bp, from which open-reading frames (ORFs) were predicted. Unassembled reads from all samples were mixed, assembled, and predicted for ORFs. After removing redundancies, a total of 2,264,311 genes were retained. To increase the sensitivity of microbial identification, we also included 7,685,872 genes from a pig fecal microbiome reference set (Xiao et al., 2016) and obtained a non-redundant gene catalog of 8,485,766 genes. After removing genes that were not mapped by any reads from any sample, 3,343,601 genes remained, which constituted the final gene catalog for subsequent analyses (Figure 2). Importantly, genes that were common between the reference set and the set from the current study were more abundant than genes unique to either set in all three intestinal sites except in ileum, where genes unique to the current study were the most abundant (Figure 2). This is likely due to the fact that the microbial composition was more similar between the reference fecal microbiome and those from cecum and colon of the current study, while ileum possessed a more distinct microbiome.

### Taxonomic Composition of Microbial Population in Three Intestinal Locations

We searched the protein-coding gene sequences against the NCBI nr protein database to classify each gene taxonomically. To obtain the TPM fragments for each classification unit, TPMs for all genes mapped to it were summed. A total of 14,178 species, 2,797 genera, 698 families, 302 orders, 142 classes, and 163 phyla can be classified in all samples combined. Based on relative abundance, the Firmicutes and Proteobacteria were the most prevalent phyla in ileum, comprising 92.9% of the ileum microbial population. The Bacteroidetes and Firmicutes dominated the microbiota in cecum and colon, accounting for >86% of the microbial populations (Supplementary Figure S2). At the genus level, the *Clostridium* (23.7%), *Clostridioides* (20.8%), and *Escherichia* (9.5%) were the most prevalent in ileum; *Prevotella* (45.9%), *Bacteroides* (10.4%), and *Treponema* (3.8%) were the most abundant genera in cecum; and *Prevotella* (28.2%), *Clostridium* (11.5%), and *Treponema* (8.2%) were the most frequent in colon (Supplementary Figure S3). At the species level, the three most abundant species were *Clostridioides difficile* (23.9%), *Escherichia coli* (10.5%), and *Clostridium* sp. *CAG:221* (7.8%) in the ileum; *Prevotella* sp. *P5-92* (5.4%), *Prevotella* sp. *P2-180* (4.9%), and *Bacteroidales bacterium* (3.2%) in the cecum; and *Prevotella* sp. *P5-92* (5.3%), *Clostridium* sp. *CAG:138* (5.1%), and *Prevotella* sp. *P2-180* (3.4%) in the colon (Supplementary Figure S4 and Supplementary Table S3). The taxonomic composition at the phylum, genus, and species levels all suggested that cecum and colon, which are components of the hindgut of pig, had more similar dominant bacteria.

### Taxonomic Diversity of Microbiome Composition Within and Across Samples

**FIGURE 3:**
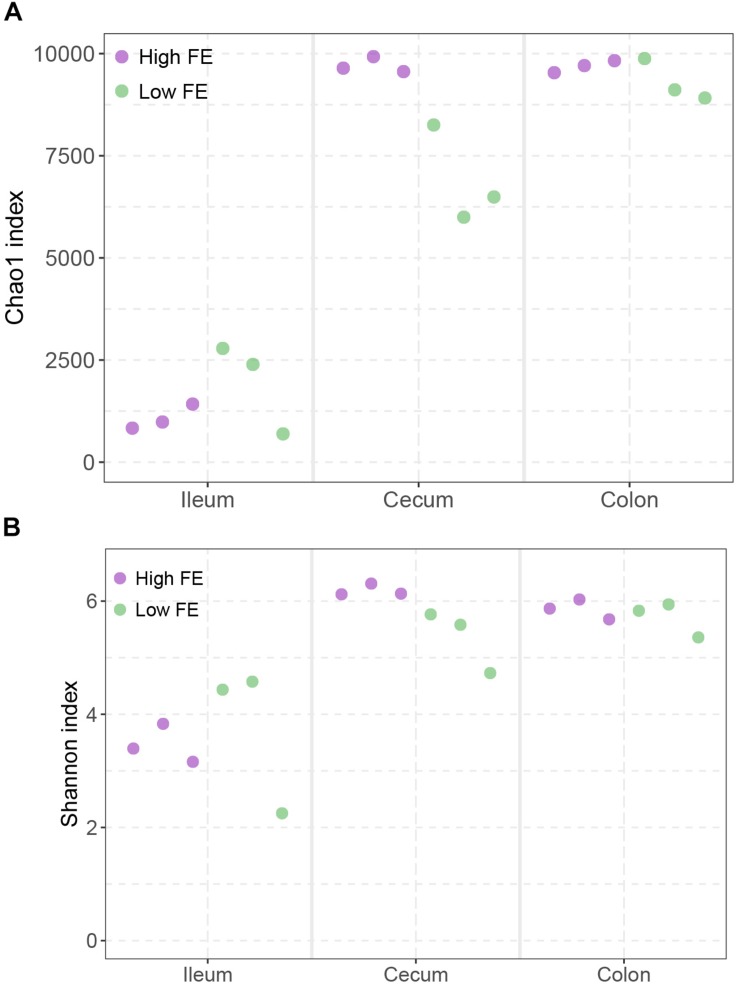
Alpha diversity of microbial community among three intestinal locations and between high and low FE groups. **(A)** The Chao1 index. **(B)** The Shannon index. Each point represents one sample as indicated by the axis label and color of the point.

To explore the community diversity of the samples, we computed two different alpha-diversity measures at the species level (Figure 3). According to the Chao1 index, which measures the richness of species, the ileum showed significantly lower richness than cecum and colon (Wilcoxon rank sum test *p* < 0.01), and only in the cecum did the high FE groups showed a slightly higher Chao1 index than the low FE group (*p* = 0.1 and Figure 3A). According to the Shannon index, which considers both the richness and evenness of species, ileums had consistently lower evenness and richness than cecum and colon (*p* < 0.01) and there was also a slightly higher Shannon index in high FE group than low FE group in cecum (*p* = 0.1 and Figure 3B).

**FIGURE 4:**
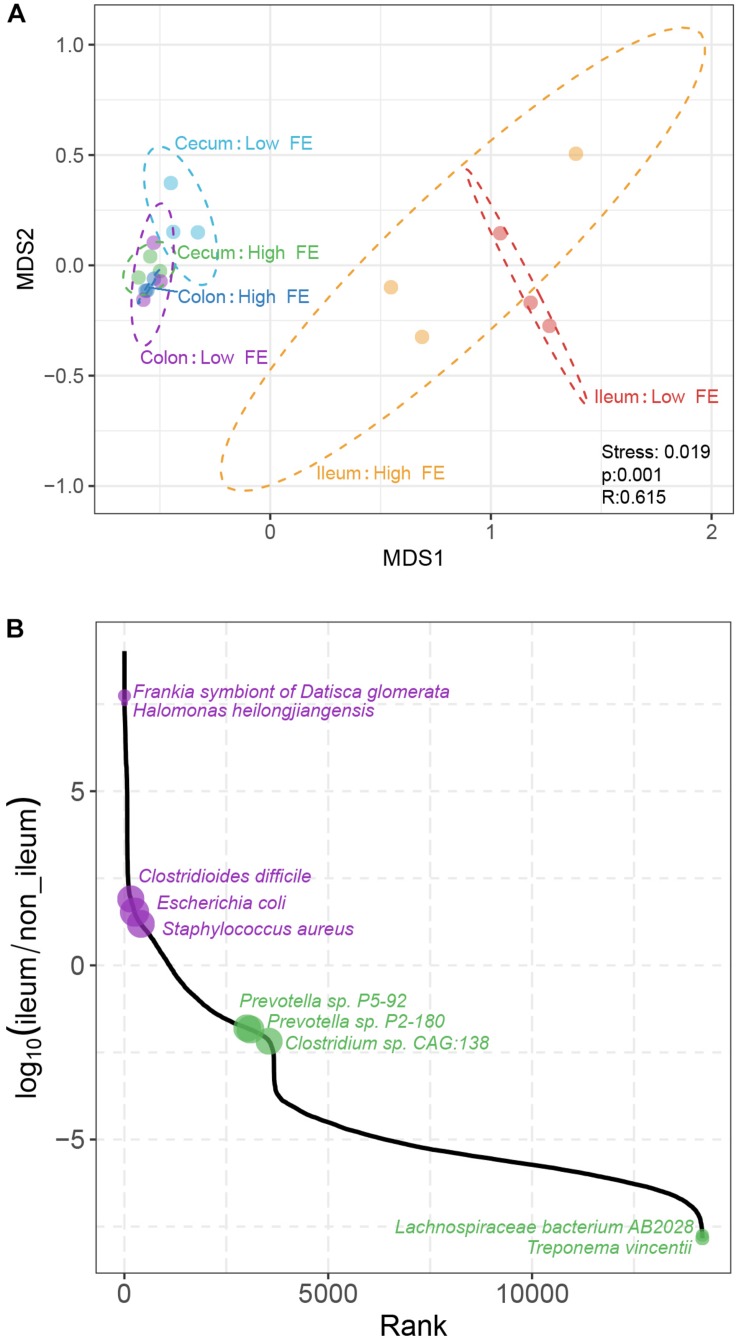
Comparison of microbial composition based on species abundance among samples. **(A)** Non-metric multidimensional scaling (NMDS) plot according to FE performance and intestinal location based on the abundance of species. The plot is based on the Bray–Curtis distances between pairs of samples. Each point represents one sample; ellipses represent the 95% confidence for all points within each cluster. Stress, the value used to estimate the NMDS ordination fitness. *R*, the statistic from the analysis of similarities (ANOSIM) that compares the mean of ranked dissimilarities between groups to the mean of ranked dissimilarities within groups. *p*, the *p*-value from ANOSIM analysis between groups. **(B)** The abundance ratio of different species between the ileum microbiota and hindgut microbiota. The species are ordered along the *X*-axis according to their rank of abundance ratio between the ileum and non-ileum.

To understand the differences in the microbiome composition between samples from different intestinal sites and FE groups, we computed the Bray–Curtis distance between pairs of samples at the species level, a beta-diversity measure. The distance matrix was visualized by NMDS. There are two apparent patterns. First, the ileum samples were clearly clustered together and separated them from the other samples. Second, globally, there is no clear separation between the high and low FE groups except in cecum, where the high FE and low FE samples were divergent in the second dimension of the NMDS plot (Figure 4A). The analysis of similarities (ANOSIM) analysis also suggested that the bacterial communities were significantly different according to intestinal location grouping (*p* < 0.05).

We identified 6,829 species whose relative abundances were significantly different between the ileum and non-ileum microbiota (*p* < 0.01, Benjamini–Hochberg *q* < 0.05, Figure 4B and Supplementary Table S4). Among them were 18 ileum specific and 4,457 non-ileum specific species, and 265 and 2,089 species that were present in both groups but more abundant in ileum and non-ileum, respectively. The large differences between the numbers of species specific to ileum versus non-ileum may be due to the low diversity of ileum microbiom, hence fewer detectable species. These results suggested that the microbial composition of the hindgut was different from the ileum, which was a component of the midgut. Among the ileum specific species, the abundances of *Frankia symbiont of Datisca glomerata* and *Halomonas heilongjiangensis* were the highest (Figure 4B). Of the non-ileum specific species, *Treponema vincentii* and *Lachnospiraceae bacterium AB2028* were the most abundant. For species that were found in all sites, the abundances of *C. difficile*, *E. coli*, and *Staphylococcus aureus* were the highest in ileum, and *Prevotella* sp. *P5-92*, *Prevotella* sp. *P2-180*, and *Clostridium* sp. *CAG:138* were the most abundant in non-ileum. To identify specific bacterial species that were characteristic of an intestinal location, we further performed LDA coupled with effect size (LEfSe) on the taxa that exhibited LDA scores greater than three. The result showed that 107 species including 72 species in non-ileum and 35 species in ileum could be potential biomarkers for midgut and hindgut distinction (Supplementary Figure S5).

**FIGURE 5:**
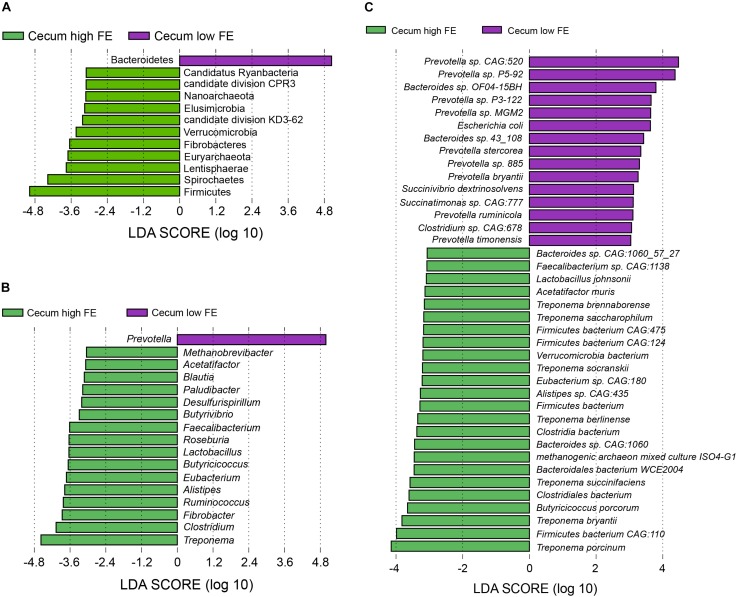
Linear discriminant analysis (LDA) effect size (LEfSe) analysis. The same analysis was performed at the **(A)** phylum, **(B)** genus, and **(C)** species levels to compare the cecum microbiota profiles between the high and low FE groups.

We also performed LEfSe analysis to identify bacteria that were different in abundance between the high and low FE groups in the cecum. In the cecum microbial population, 11 phyla including Firmicutes, Spirochaetes, Lentisphaerae, and Euryarchaeota in the high FE group and Bacteroidetes in the low FE group were characteristic of the respective FE groups (Figure 5A). At the genus level, 16 genera (nine of them belong to *Clostridiales*) including *Treponema*, *Clostridium*, *Fibrobacter, Ruminococcus*, and *Lactobacillus* in the high FE pigs and only *Prevotella* in the low FE pigs could be considered potential biomarkers in the cecum for the different FE groups (Figure 5B). At the species level, 15 species in the low FE group, including 9 species from the *Prevotella* genus, and 24 species in the high FE group that included 7 species from *Treponema*, 4 species from *Firmicutes bacterium*, and 3 species from *Bacteroides* could be potential biomarkers in cecum for different FE (Figure 5C and Supplementary Table S5).

### Functional Annotation of Microbiomes

**FIGURE 6:**
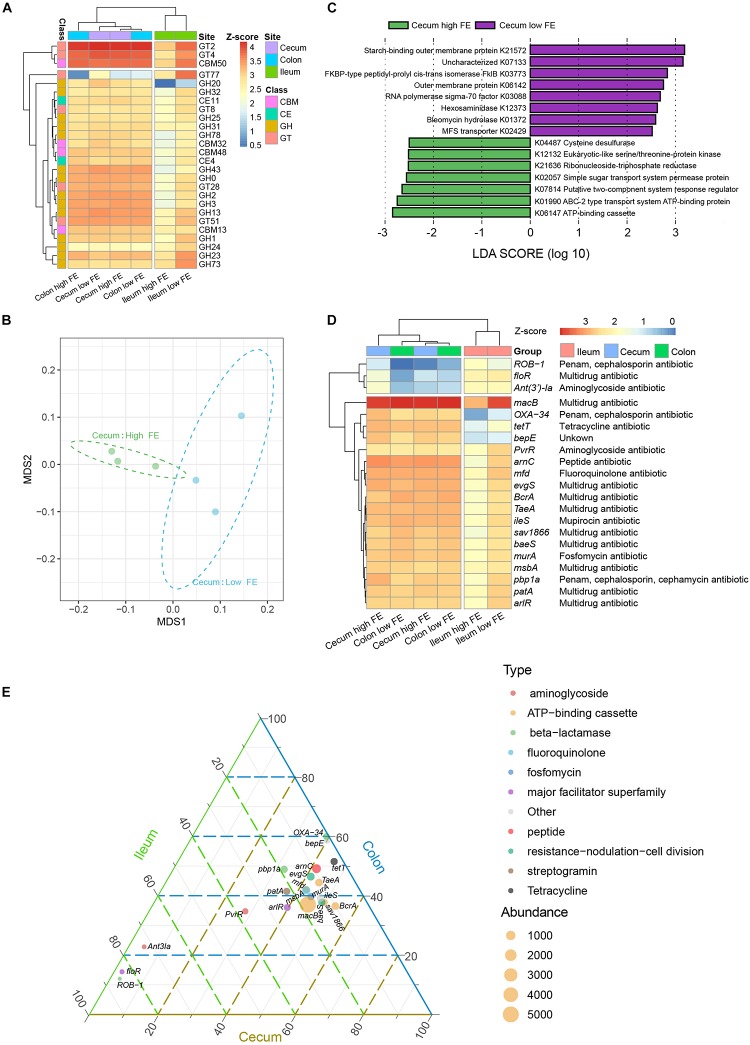
Functional annotation of the microbiomes. **(A)** Heatmap of the 10 most abundant (based on TPM value) carbohydrate active enzyme (CAZy) families in any of the groups. Color scale shows the abundance of CAZy enzyme family within each group. *Z*-score, calculated with the formula *z* = (*x* − μ)/σ, where *x* is the log10 of abundance of enzymic families in each group, μ is the mean value of the log10 of abundance in all groups, and σ is the standard deviation of the log10 of abundance. **(B)** NMDS plot of high and low FE of cecum samples based on the abundance of CAZy families. **(C)** LEfSE analysis for KEGG orthology (KO) to compare the cecum microbiota functional profiles between the high and low FE group. **(D)** Heatmap of the 10 most abundant (based on TPM value) antibiotic resistance genes (ARGs) in any of the groups. **(E)** Ternary plot showing the abundance comparison of the 10 most abundant ARGs of each group in the ileum (green), cecum (brown), and colon (blue). The sum of the abundance for one specific ARG in these three types of gut was set as 100%. The percentage (%) of one specific ARG in each gut location is equal to its corresponding abundance divided by the abundance sum of this ARG in the three intestinal locations. The symbol size indicate the total abundance of the ARGs. The symbol color indicated the types of the ARGs.

To gain functional insights into the diversity of the microbiomes, we annotated the genes based on three functional databases. First, sequences in the final gene catalog were aligned to protein sequences in the CAZy database. We were able to classify the sequences into six enzyme classes and 314 families. The glycoside hydrolases (GHs) and glycosyl transferases (GTs) were dominant in all intestinal locations (Supplementary Figure S6). At the enzyme family level, we found glycosyltransferase 2 (GT2) and carbohydrate-binding module 50 (CBM50) were the most abundant in the ileum, GT2, GT4, and CBM50 were the most abundant in the cecum and colon (Figure 6A). Based on their Bray–Curtis distance, the abundances of CAZy enzyme families clustered the samples into two groups according to their intestinal locations, suggesting that microbiota in the colon and cecum share similar carbohydrate enzyme profiles. Similar to taxonomic composition, the enzymatic composition among the ileum samples was notably more variable than that among cecum and column (Supplementary Figure S7). We saw no clear separation between the high and low FE groups, which could be obscured by the large variability among the ileum samples and between the ileum and the non-ileum samples (Supplementary Figure S7). While several enzyme families were significantly different between ileum and non-ileum samples based on Wilcoxon test, we did not find any enzyme showing significant difference between the high and low FE groups at any gut location (Supplementary Table S6).

When the gene catalog was aligned to the KEGG database, 6,462 KOs were identified and assigned to 365 KEGG pathways. All samples could be clustered into ileum and non-ileum patterns based on their KOs abundance (Supplementary Figure S8). We further used LEfSe to identify potential biomarkers separating the functional characteristics between the ileum and non-ileum (cecum and colon). At a cutoff of LDA score = 3, 20 KOs showed significant differences (Supplementary Figure S9). We found that the functional characteristics of the non-ileum sites were dominated by signaling and cellular processing and carbohydrate metabolism, which was consistent with the CAZy annotation, and that the ileum was specialized in genetic information processing and nucleotide metabolism. Based on the abundances of KOs, only samples from the high and low FE groups in the cecum can be separated clearly (Figure 6B). The LEfSe analysis showed that 15 KOs could be considered potential biomarkers in the cecum for the different FE groups (Figure 6C and Supplementary Figure S10). The KOs more abundant in the low FE group were associated with genetic information processing (K03773, K03088), amino sugar and nucleotide sugar metabolism (K12373), and transport system (K21572, K02429). The KOs more abundant in the high FE group were associated with the metabolism of protein, nucleotide, cofactors, and vitamins (K12132, K21636, and K04487), and monosaccharide or energy transportation (K02057, K01990, and K06147) (Supplementary Figure S10).

Many coexisting microbes can produce a variety of antibiotics or bacteriocins to inhibit the growth of other microbes when competing for the nutrients in intestinal digesta (Hong et al., 2016). We analyzed the genes by the CARD database to identify and compare the abundances of antibiotic resistance genes (ARGs). Figure 6D shows the 10 most abundant ARGs in any intestinal sites, which clustered the samples into an ileum cluster and a non-ileum cluster. This suggested that the microbe-contributed ARGs were different between ileum and hindgut (cecum and colon). The ATP-binding cassette antibiotic efflux pump genes including *macB*, *BcrA*, *TaeA*, and *sav1866* participate in utilizing the free energy of ATP hydrolysis to expel multiple antibiotics from the cells against their concentration gradient (Lubelski et al., 2007). These genes were much higher in abundance in cecum and colon digsta than in ileum. Based on the abundance of ARGs in each sample, only samples from the high and low FE groups in the cecum can be separated clearly (Supplementary Figure S11). The LEfSe analysis showed that there were 25 ARGs that contributed to the differences among the high and low FE groups in cecum (Supplementary Figure S12), and the *macB* was the most abundant (Supplementary Figure S13). We also found that species in the *Prevotella* genus were the biggest contributors of *macB* gene in the cecum low FE group, while species in the *Treponema* and *Clostridium* genera were among the top contributors in the cecum high FE group (Supplementary Figure S14). At the species level, *Phascolarctobacterium succinatutens*, *Treponema porcinum*, and *Treponema bryantii* were the biggest contributors of *macB* in the cecum high FE group, and *Prevotella* sp. *CAG:520* and *Prevotella* sp. *P5-92* were the biggest contributors in the cecum low FE group (Supplementary Table S7 and Supplementary Figure S14). These results were mostly consistent with the potential species biomarkers in the cecum for the different FE groups. It implied that the *macB* gene played important roles in regulating microbial composition, which might affect the FE of pigs.

## Discussion

The intestinal microbiota play a crucial role in nutrient processing and the harvesting of ingested energy in pigs (McCormack et al., 2017) and other mammals (Ley et al., 2006). It is plausible to suggest that the pig gut microbiota could potentially affect their growth performance, such as FE. In recent years, many studies have focused on this thesis (McCormack et al., 2017; Tan et al., 2017; Yang et al., 2017; Quan et al., 2018, 2019). However, these studies were limited by either a single sampling site or a low-resolution sequencing method. This study is one of the firsts to perform whole metagenome sequencing of samples from multiple intestinal regions (ileum, cecum, and colon) of DLY pigs that exhibited high and low FE. To increase the sensitivity of microbial identification, we also combined a pig metagenomic reference gene catalog developed from a large-scale effort (Xiao et al., 2016).

Consistent with previous studies (Looft et al., 2014), the dominant phyla in pig ileum were Firmicutes and Proteobacteria, whereas the phylum-level compositions of the cecum and colon were highly similar and both harbored dominant Bacteroidetes and Firmicutes (Supplementary Figure S1). However, we still found that prevalent genera or species that were different between previous studies and the present study. Yang et al. (2016) found that *Clostridium*, *SMB53*, and *Escherichia* were the most abundant genera in ileum and *Prevotella*, *Escherichia*, and *Lactobacillus* were the most abundant in cecum of Laiwu pigs. Xiao et al. (2018) revealed that *Lactobacilli* and *Clostridia* were the two most abundant genera in the ileum, cecum, and colon of Landrace pigs. However, our data showed that the most abundant genera in ileums were *Clostridium*, *Clostridioides*, and *Escherichia*. But *Prevotella*, *Bacteroides*, and *Treponema* were the most abundant genera in the cecum and colon. This highly variable microbial community composition at the lower taxonomic level may be influenced by multiple factors, such as breed, age, or feed, which were different between studies. Additionally, we also found that intestinal location is the major determinant of taxonomic and functional profiles likely because of their different ecological environments and physiologies. For example, when considering the functions of the different species between ileum and hindgut (cecum and colon), we found that *Prevotella* sp. *P5-92*, *Prevotella* sp. *P2-180*, and *Clostridium* sp. *CAG:138* were more abundant in the hindgut. These bacteria were associated with polysaccharide metabolism (Bekele et al., 2011; Wu et al., 2011), which was consistent with the physiological capacity of the large intestinal (Serena et al., 2008).

Only the cecum samples showed appreciable separation for the high and low FE groups in a dimension reduction plots using both taxonomic and functional profiles. The cecum is an important site for bacterial fermentation of dietary fiber, which is associated with the utilization of feed nutrient and energy (Zhang et al., 2013; He et al., 2018), especially when pigs are fed with high fiber feed. Therefore, the different fermentation capacities of the microbiomes could be a crucial factor in determining FE performance. The Firmicutes and Bacteroidetes were characteristic biomarkers of the high and low FE groups at the phylum level (Figure 5). Indeed, previous studies suggested that the Firmicutes was important in energy harvesting of mice (Turnbaugh et al., 2006), and an increased Firmicutes in pigs intestinal might be able to improve fatness (Yang et al., 2016). Consistent with a previous study in humans, where Bacteroidetes was relatively higher in abundance in lean individuals compared to obese individuals (Ley et al., 2006), lower abundance of Bacteroidetes in gut microbiota was found in the high FE pigs and higher Bacteroidetes was found in the low FE pigs. The Euryarchaeota, which is a phylum of archaea, was more abundant in high FE pigs group. The Euryarchaeota included many Methanogens that can produce methane through cellulose metabolism in hypoxic conditions, such as the ruman of ruminants (Lin and Miller, 1998).

*Prevotella* was considered a polysaccharide-degrading bacterial genus (Lamendella et al., 2011). Consistent with a previous study, we also found that the *Prevotella* was a feature biomarker of the cecal microbiota in low FE pigs (Tan et al., 2017). In general, higher *Prevotella* relative abundance was associated with high carbohydrate diet. However, the *Prevotella* was also antagonistic with other microbes such as *Bacteroides*, which was associated with the protein breakdown process (Ley, 2016; Coelho et al., 2018). Excessive *Prevotella* may hinder the generation of a high efficiency nutrient harvesting community especially when the animals were feed compound feed such as in this study.

We identified feature biomarkers of the cecal microbiota in high FE pigs including the genera *Fibrobacter* and *Ruminococcus*, which were associated with the degradation of plant-based cellulose in mammalian gut (Koike and Kobayashi, 2001; Flint, 2004). In addition, the genus *Lactobacillus* was also enriched in high FE cecum. *Lactobacillus* was the member of lactic acid bacteria (LAB), many species such as *Lactobacillus johnsonii* can be used as a probiotic, which is able to perform fermentation and produce lactic acid (Ward and Timmins, 1999; Pridmore et al., 2004). The *Lactobacillus reuteri*, a species of *Lactobacillus*, has been linked to obesity and weight gain in children. The *E. coli*, a species in the Proteobacteria phylum, has been linked with the absence of obesity in children. These results were consistent with our findings that low FE pigs had more abundant *E. coli* and high FE pigs had more abundant *Lactobacillus* (Million et al., 2013a, b). Furthermore, many species from the *Lactobacillus* genus were associated with weight gain in animals and humans (Million et al., 2012). The members of the genus *Treponema* can exist as both pathogens and commensals (Rosewarne et al., 2012). In the present study, seven species in *Treponema* were more abundant in the cecal microbiota of high FE pigs. They mainly participate in the carbohydrate digestion in swine intestine (Cwyk and Canaleparola, 1979; Stanton and Canaleparola, 1980; Paster and Canaleparola, 1985; Nordhoff et al., 2005). Furthermore, three species under the *Bacteroides* genus were high FE group biomarkers. This genus dominated the human gut with protein-rich diet and was associated with the protein breakdown process (Gorvitovskaia et al., 2016). Nine cellulolytic species from the *Prevotella* genus were biomarkers of cecal microbiota in high FE pigs. A previous study suggested that the abundances of *Bacteroides* and *Prevotella* might be anticorrelated in mouse intestine because they were antagonistic (Ley, 2016). In addition, previous studies (Schwab et al., 2014; Magnusson et al., 2015) in mice found that the order Clostridiales, which included nine genera biomarkers of high FE groups in our study, had a negative relationship with the order Bacteroidiales, which includes the *Prevotella* genus. High abundance of Bacteroidiales accompanied with a low abundance of Clostridiales that can reduce inflammation (Schwab et al., 2014), while increased Clostridiales and decreased Bacteroidetes could be beneficial to fit high-energy diet (Magnusson et al., 2015).

Future studies are needed to investigate the complex interactions among the bacteria, which establish the different microbiome profiles and affect dietary feed utilization and energy harvest in the high and low FE groups. The KEGG functional annotation results confirmed this point, the high FE group has more abundant of KOs related to the metabolism of protein, nucleotide, cofactors and vitamins, and monosaccharide or energy transportation (Figure 6). Forthermore, in the Yang et al. (2017) study, they found that KOs related to nitrogen metabolism, amino acid metabolism, and transport system were positively associated with porcine FE. Tan et al. (2017) also obtained similar result that microbiota affect host FE mainly through the transport pathways of multiple physiological substrates, such as lysine, glycan, and ornithine, which can participate in the synthesis of many proteins. Furthermore, when we traced the source of the most abundant ARG *macB*, we found that the *Prevotella* sp. and *Treponema* sp. were the largest contributors in the low and high FE groups, respectively. Previous bioinformatic analysis revealed that the *macB* gene was widespread throughout bacterial genomes (Khwaja et al., 2005). In addition, the *macB* gene was suggested to be related to the utilization of the free energy of ATP hydrolysis to expel multiple antibiotics from the cells against their concentration gradient (Lubelski et al., 2007). Taken together, the *macB* gene can affect the physiological functions and energy metabolism of microbes. It is possible that the ARG *macB* may play important roles in regulating microbial composition and further affecting FE of the hosts by generating a more efficient nutrient harvesting microbioal community.

However, due to limitation of the sample size, this study was underpowered to uncover abundance differences that are of smaller magnitudes and interactions between species. The choice of the particular sample size was based on several factors, including selecting animals that were maximally divergent in their FE phenotypes and microbial composition, sequencing fewer animals with high depth and incorporating reference microbiomes to increase sensitivity. Nevertheless, this study did identify a small number of potential biomarkers in cecum microbiota at species resolution that can be followed up in future studies in an attempt to improve FE in pigs.

## Conclusion

In conclusion, we found that the cecum and colon have similar microbial taxonomic composition and function, and have higher ability in polysaccharide metabolism than the ileum. Cecum microbiota in high FE pigs have slightly higher richness and evenness than low FE pigs. Furthermore, we identified 12 phyla, 17 genera, and 39 species that were potentially associated with FE in cecum microbiota. These species in the cecum of the high FE pigs have a greater ability to utilize dietary polysaccharides and proteins. We found evidence that the *macB* antibiotic resistant gene might play an important role in construting microbial community. In particular, bacteria from the genus *Prevotella* might impair the establishment of a more effective nutrition harvesting microbiota because the interaction between them and other benefical microbes. However, the exact mechanisms remain unclear, thus warranting further investigation. These findings improve our understanding of the microbiotal compositions in the different gut locations of DLY pigs, identify many biomarkers and provide important insights into improving FE in pigs in the future.

## Data Availability Statement

Publicly available datasets were analyzed in this study. This data can be found here: PRJEB11755, http://gigadb.org/dataset/view/id/100187/token/F4CDHYruxob OKmsE.

## Ethics Statement

The animal study was reviewed and approved by the Animal Care and Use Committee (ACUC) of South China Agricultural University (SCAU).

## Author Contributions

JY, WH, and ZW conceived of and designed the experiments, and revised the manuscript. JQ, YY, LP, JW, DR, YQ, RD, XW, EZ, and JY performed the experiments and collected the samples. JQ and WH analyzed the data and wrote the manuscript. ZW and GC contributed the materials. All authors reviewed and approved the manuscript.

## Conflict of Interest

The authors declare that the research was conducted in the absence of any commercial or financial relationships that could be construed as a potential conflict of interest. The reviewer WR declared a shared affiliation, with no collaboration, with the authors to the handling Editor at the time of review.

## Footnotes

1 https://github.com/cjfields/readfq

2 https://maple.jamstec.go.jp/maple/maple-2.3.1/

## Supplementary Material

FIGURE S1 Flow chart depicting data processing.

FIGURE S2 Top 10 relative abundance bacterial phyla present in each group.

FIGURE S3 Top 10 relative abundance bacterial genera present in each group.

FIGURE S4 Top 10 relative abundance bacterial species present in each group.

FIGURE S5 Species characteristic of the ileum and hind-gut microbial community.

FIGURE S6 The abundance of six classes of carbohydrate-active enzymes in each intestinal location.

FIGURE S7 NMDS plot of high and low FE groups at each gut site based on the abundance of CAZy families.

FIGURE S8 NMDS plot of high and low FE groups at each gut site based on the abundance of KOs.

FIGURE S9 The KOs and their description that can be characteristic of the ileum and hind-gut microbial community.

FIGURE S10 The heatmap showing the KO biomarker abundance of the cecum samples.

FIGURE S11 NMDS plot of high and low FE of cecum samples based on the abundance of ARGs.

FIGURE S12 ARGs characteristic of the high and low FE groups in cecum microbial community.

FIGURE S13 The abundance of ARG biomarkers of the high and low FE groups in cecum microbial community.

FIGURE S14 The relative abundance of top 10 abundant contributors of *macB* resistant gene between the high and low FE groups in cecum microbiota.

TABLE S1 The phenotype value of experimental animal.

TABLE S2 Summary of metagenomic sequencing data.

TABLE S3 The microbiota composition of three gut locations at phylum, genus, and species levels.

TABLE S4 The significantly different species between ileum and hindgut group.

TABLE S5 The LDA score of potential biomarkers in the cecum for the different FE groups at phylum, genus, and species levels.

TABLE S6 The significantly different enzymes between ileum and hindgut group.

TABLE S7 Top 20 contributors of macB gene at genus and species levels.
