# Supplementary figures and images for: Metagenomic Characterization of Intestinal Regions in Pigs With Contrasting Feed Efficiency
Source: Front Microbiol. 2020 Jan 23;11:32. doi: 10.3389/fmicb.2020.00032 (PMC6989599; doi:10.3389/fmicb.2020.00032)

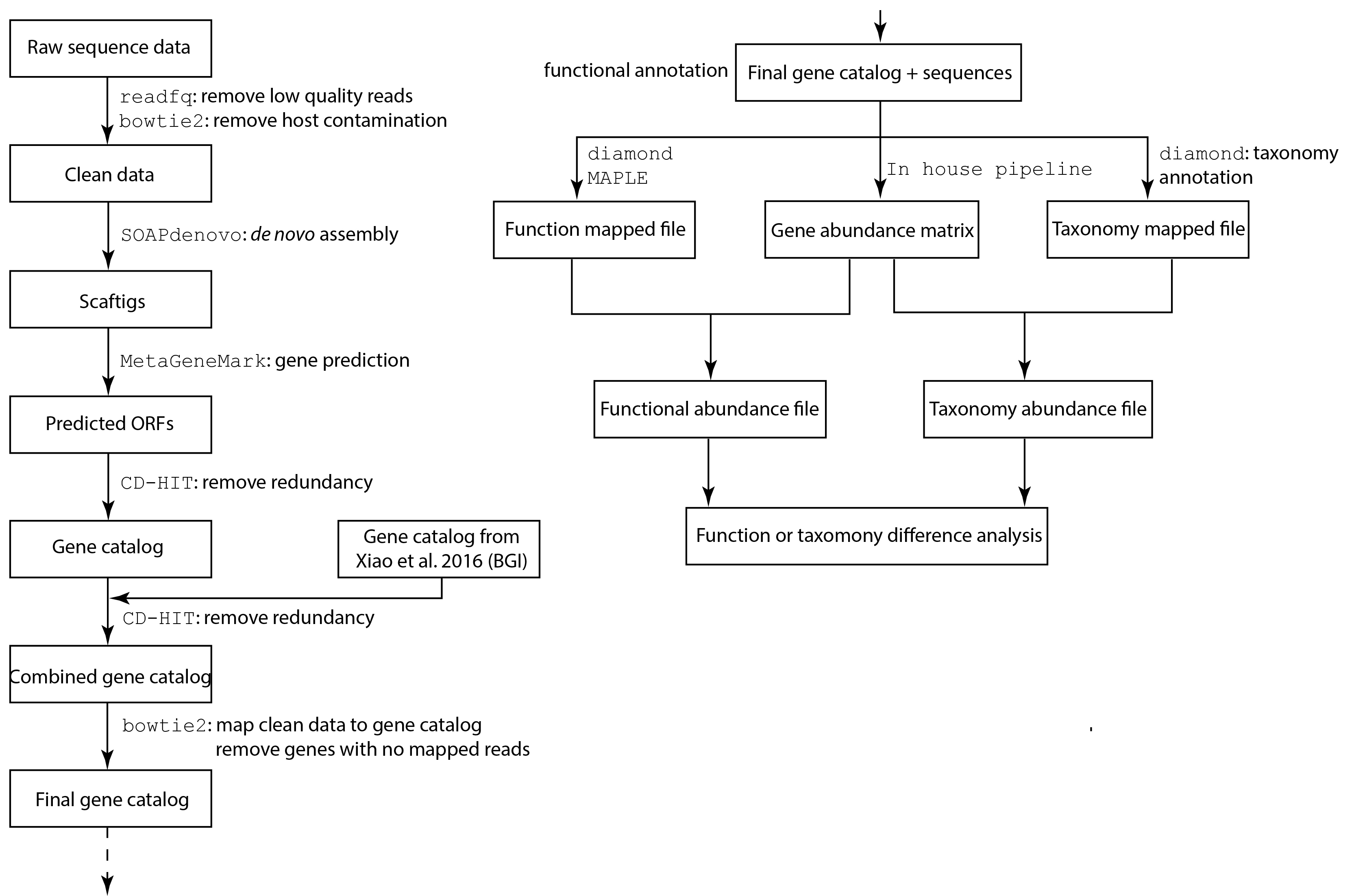

Supplement: FIGURE S1 — Flow chart depicting data processing. [file Image_1.JPEG]

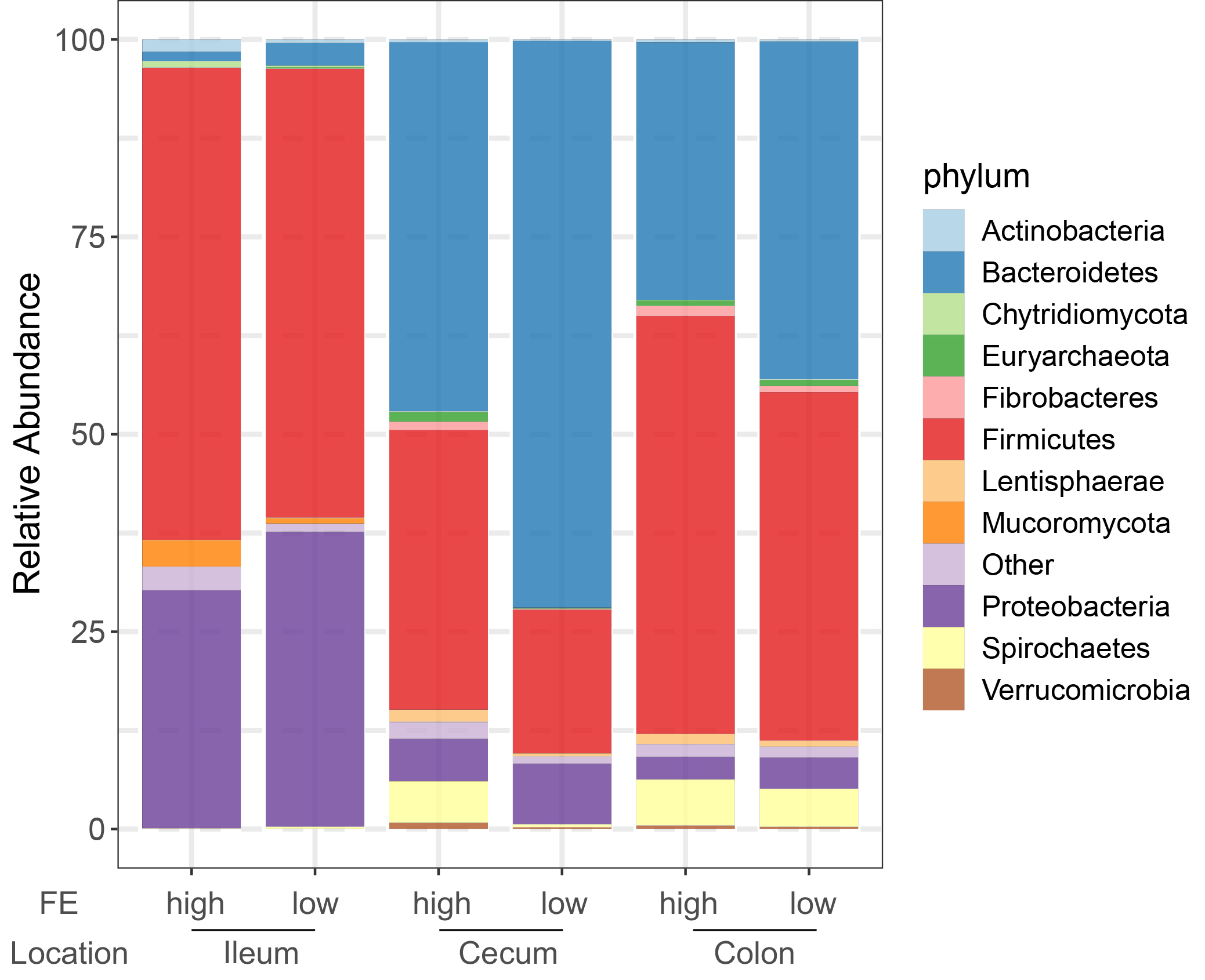

Supplement: FIGURE S2 — Top 10 relative abundance bacterial phyla present in each group. [file Image_2.JPEG]

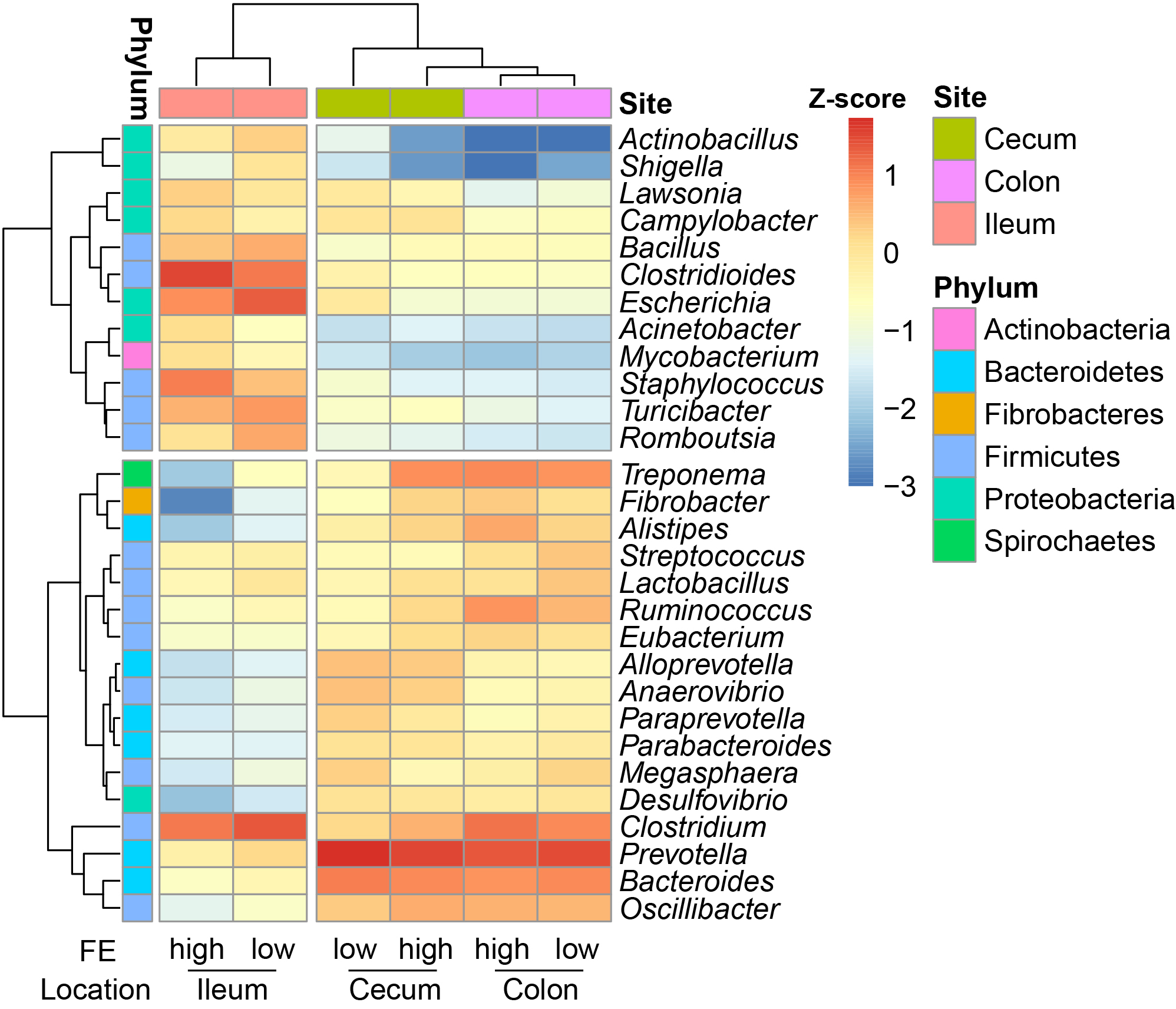

Supplement: FIGURE S3 — Top 10 relative abundance bacterial genera present in each group. [file Image_3.JPEG]

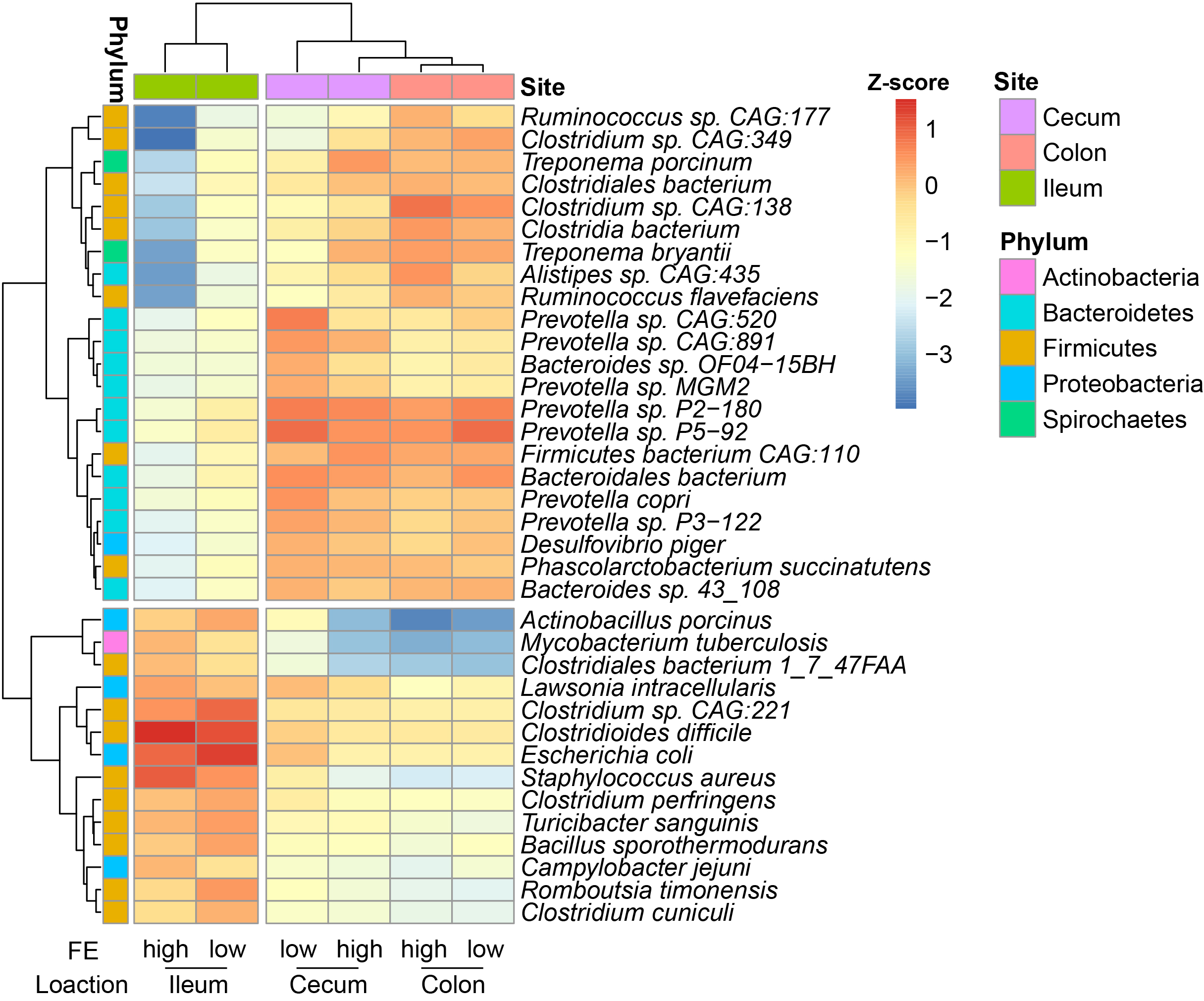

Supplement: FIGURE S4 — Top 10 relative abundance bacterial species present in each group. [file Image_4.JPEG]

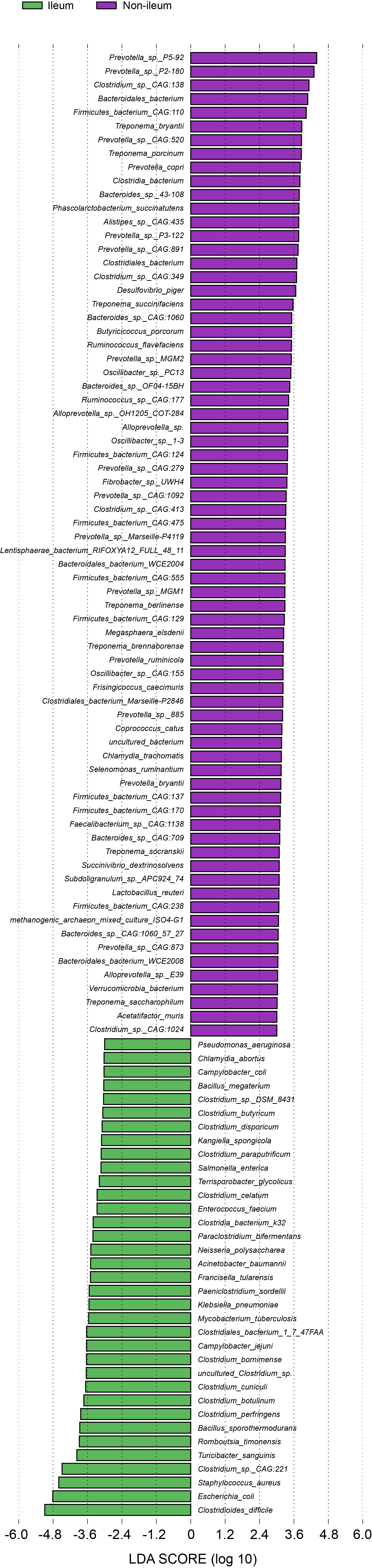

Supplement: FIGURE S5 — Species characteristic of the ileum and hind-gut microbial community. [file Image_5.JPEG]

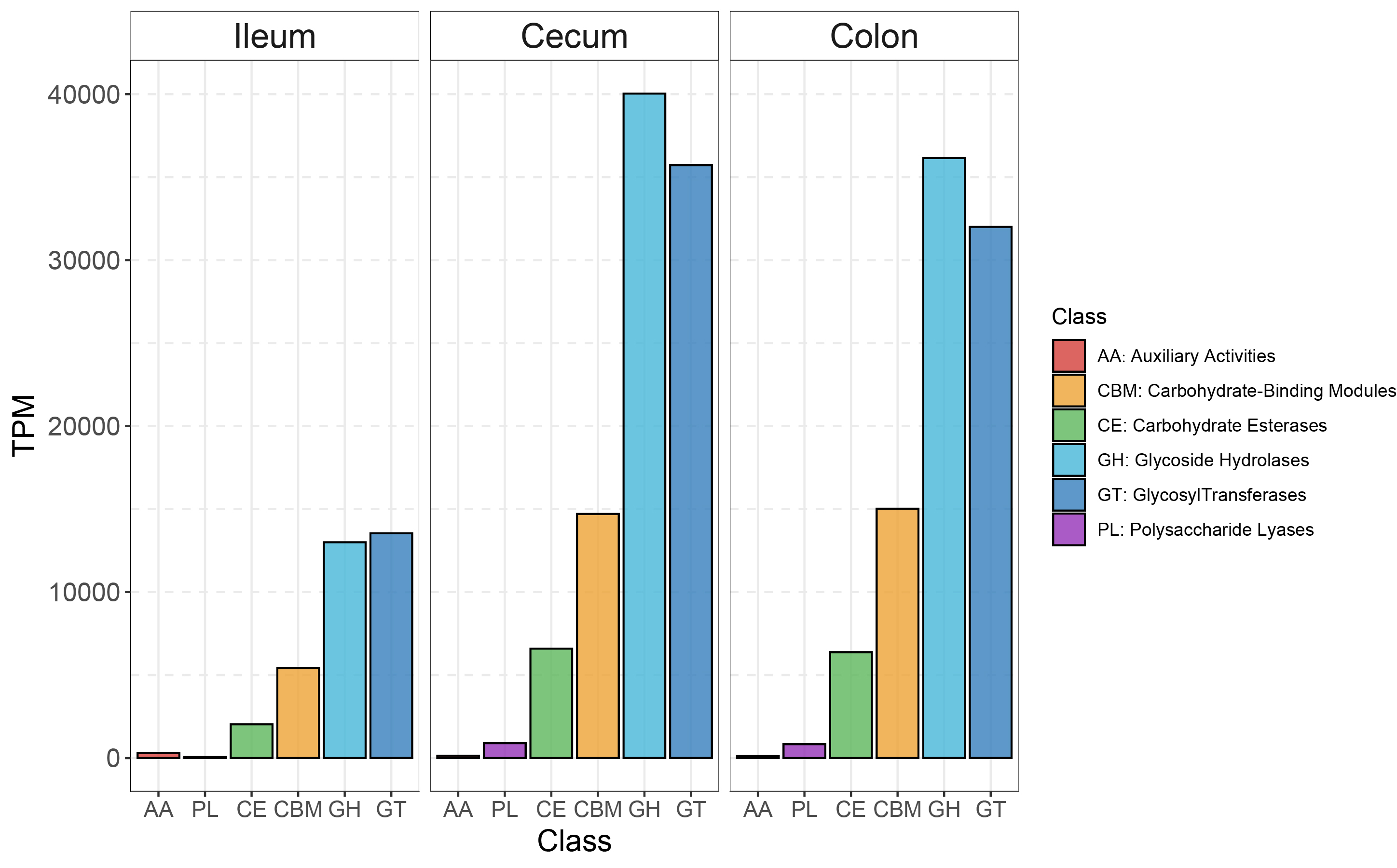

Supplement: FIGURE S6 — The abundance of six classes of carbohydrate-active enzymes in each intestinal location. [file Image_6.JPEG]

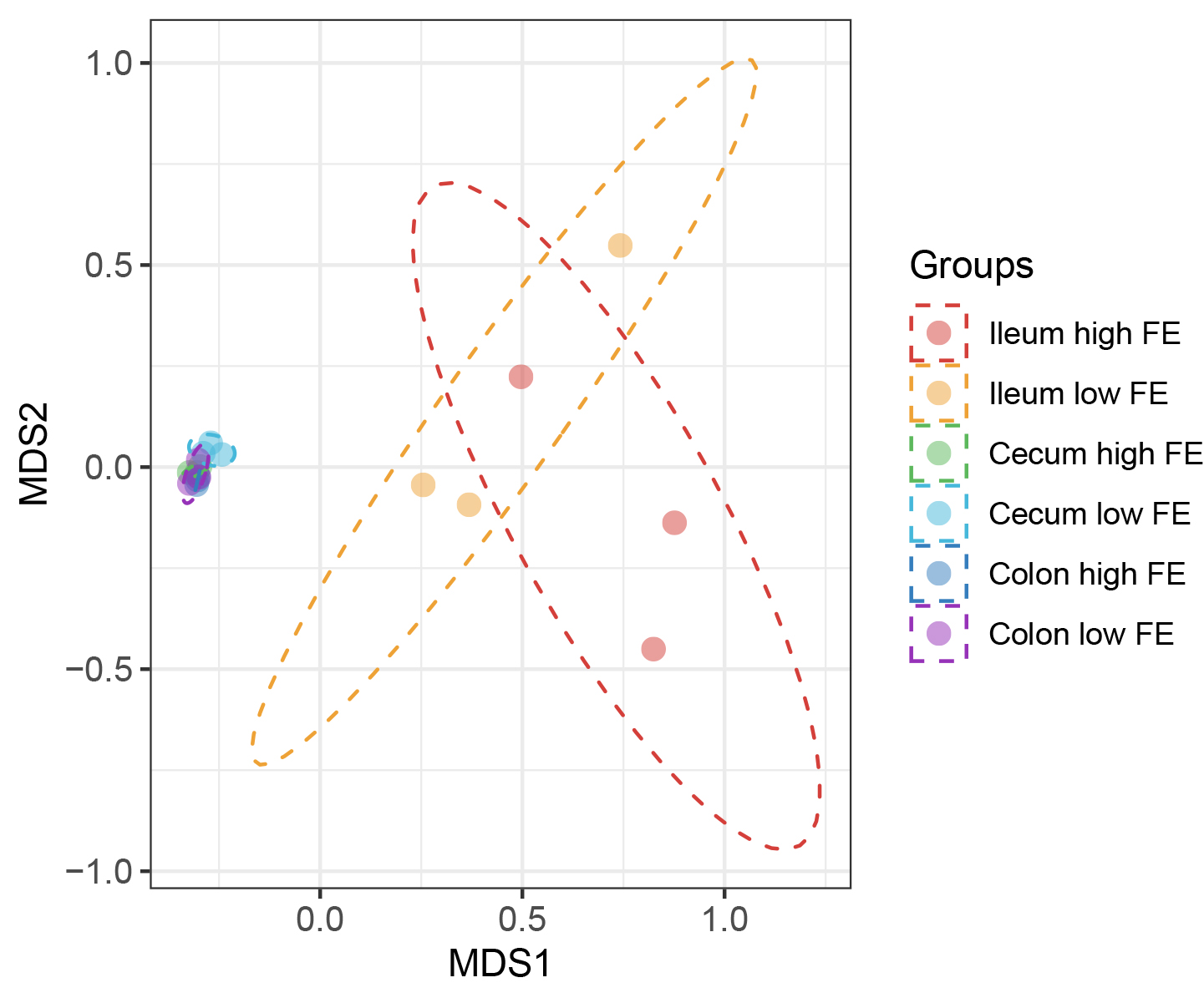

Supplement: FIGURE S7 — NMDS plot of high and low FE groups at each gut site based on the abundance of CAZy families. [file Image_7.JPEG]

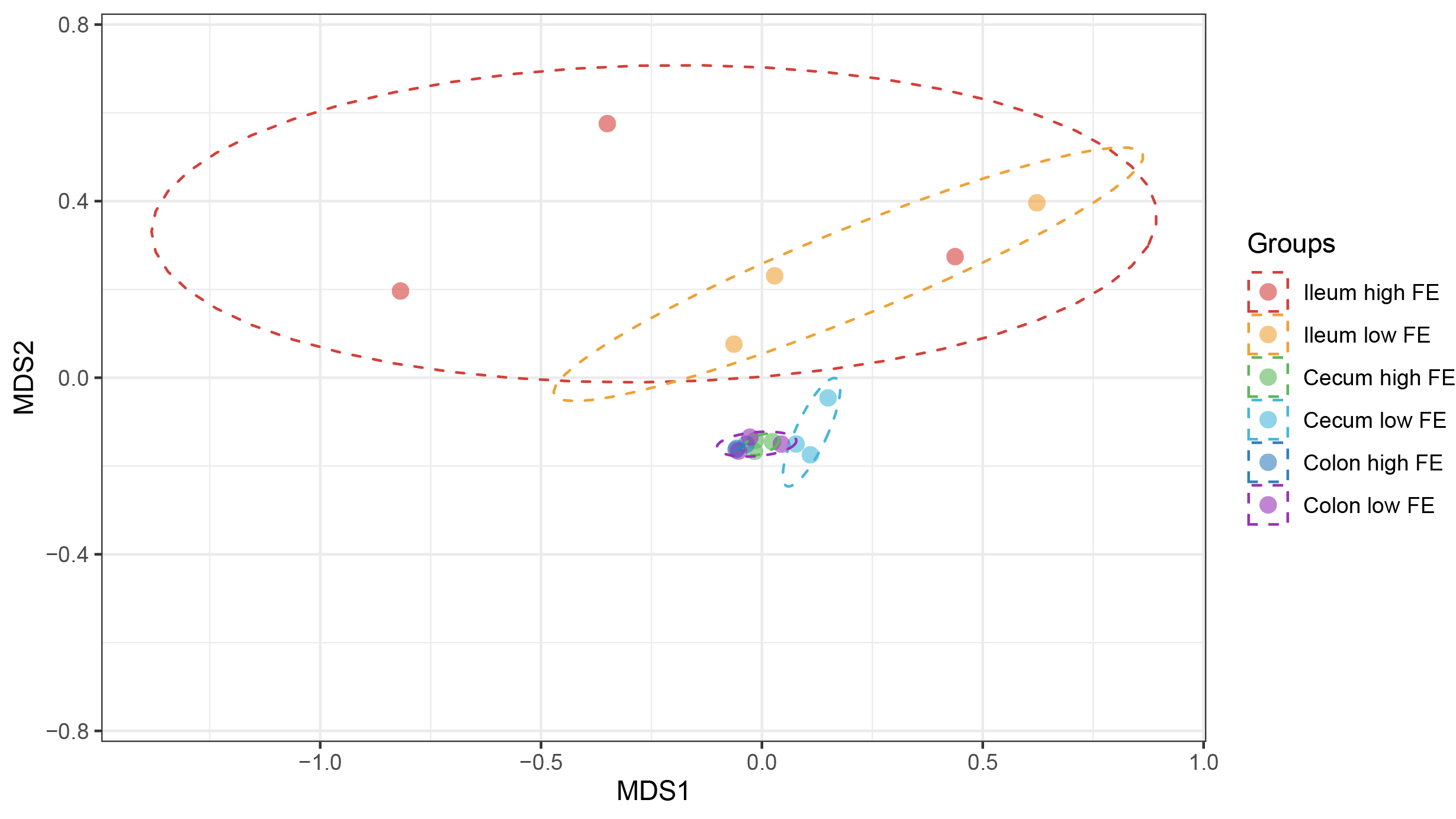

Supplement: FIGURE S8 — NMDS plot of high and low FE groups at each gut site based on the abundance of KOs. [file Image_8.JPEG]

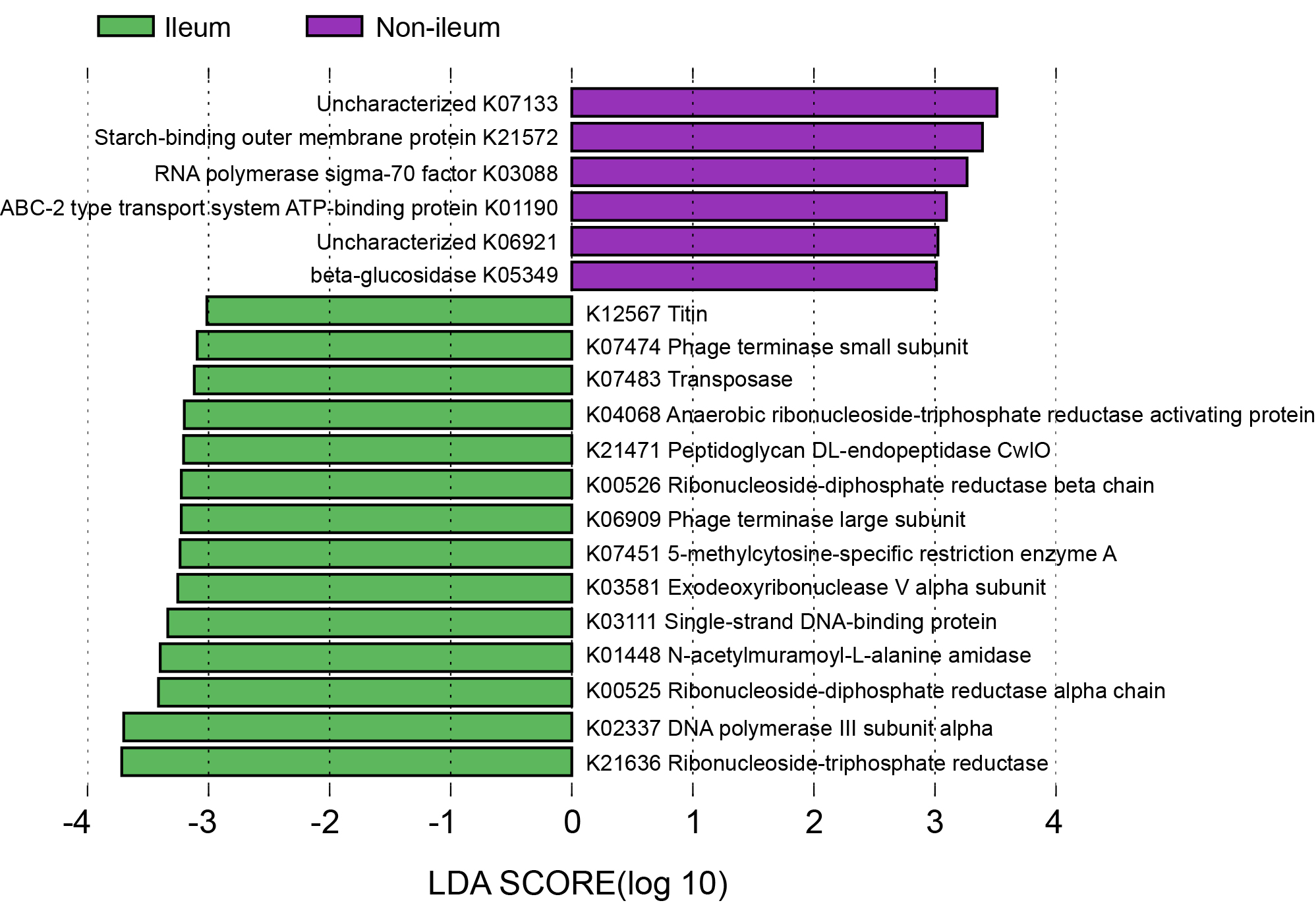

Supplement: FIGURE S9 — The KOs and their description that can be characteristic of the ileum and hind-gut microbial community. [file Image_9.JPEG]

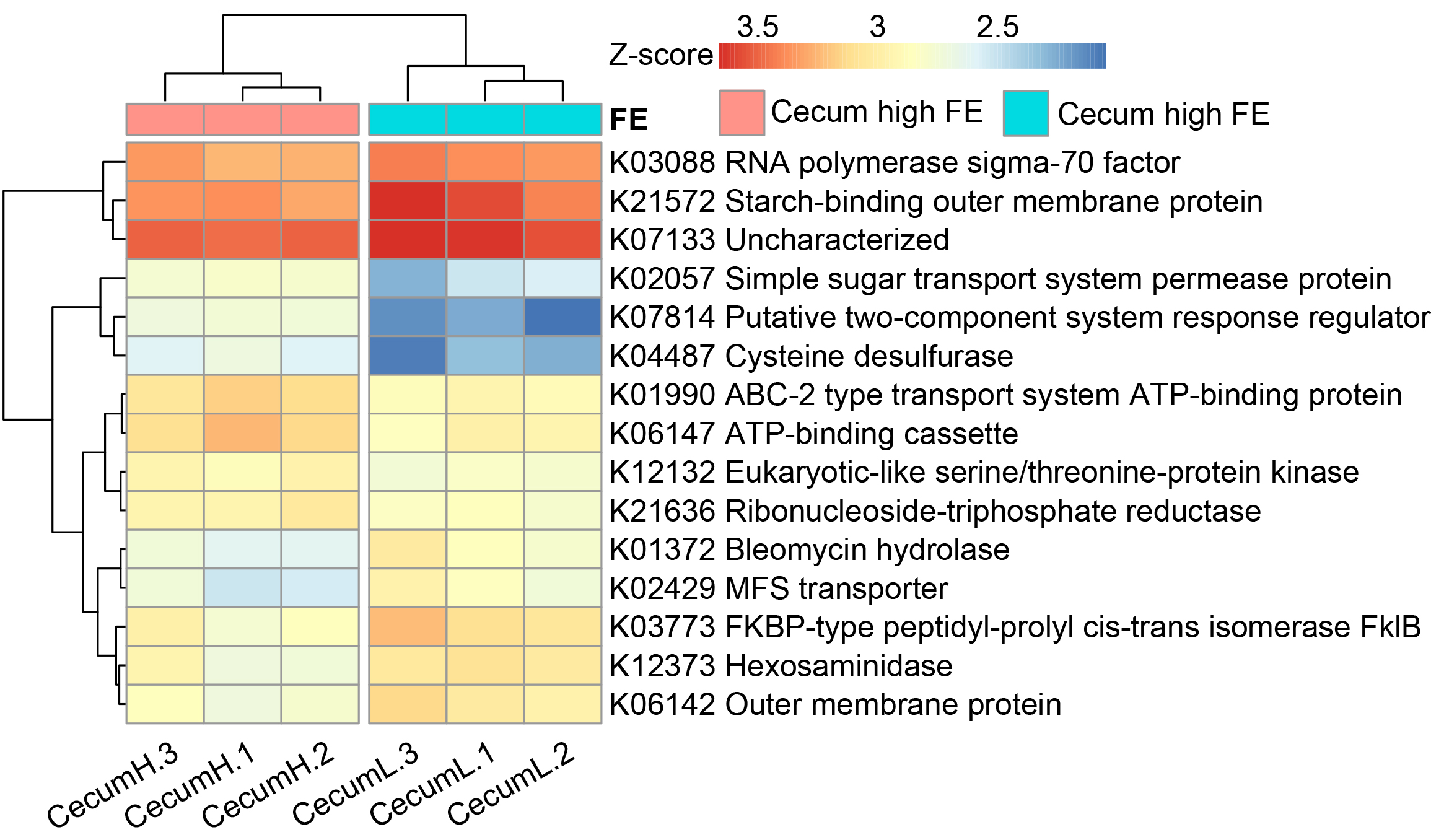

Supplement: FIGURE S10 — The heatmap showing the KO biomarker abundance of the cecum samples. [file Image_10.JPEG]

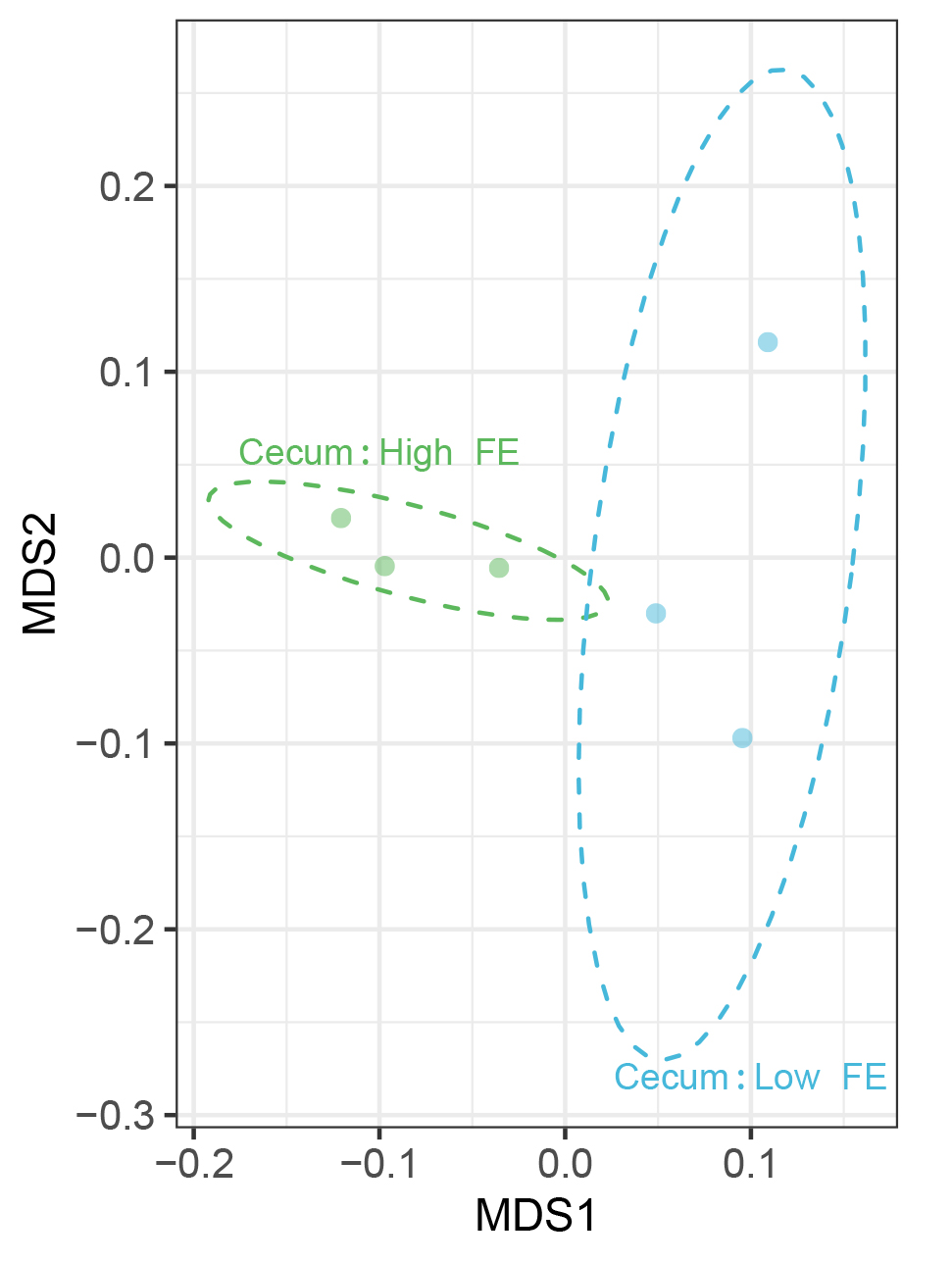

Supplement: FIGURE S11 — NMDS plot of high and low FE of cecum samples based on the abundance of ARGs. [file Image_11.JPEG]

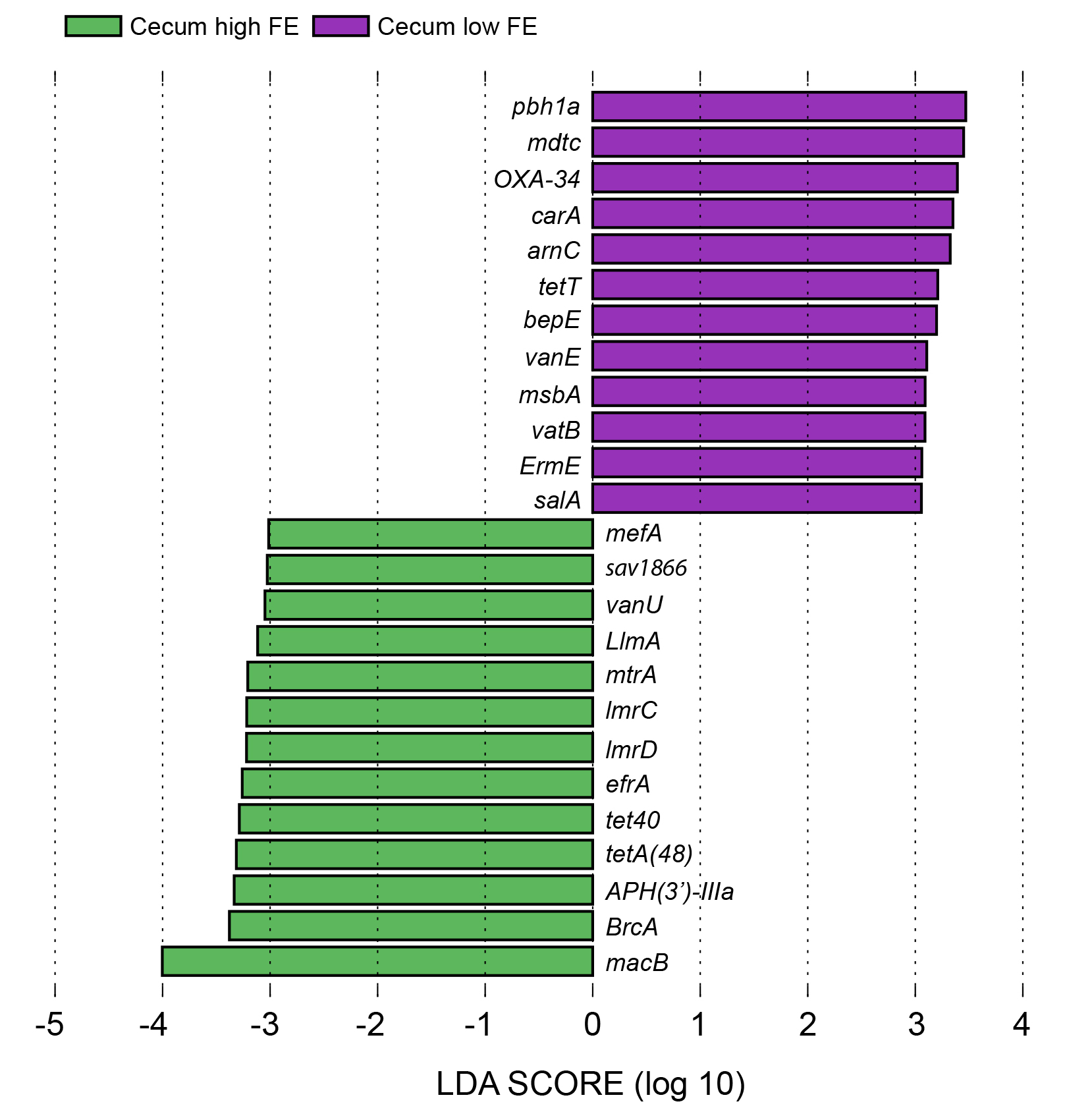

Supplement: FIGURE S12 — ARGs characteristic of the high and low FE groups in cecum microbial community. [file Image_12.JPEG]

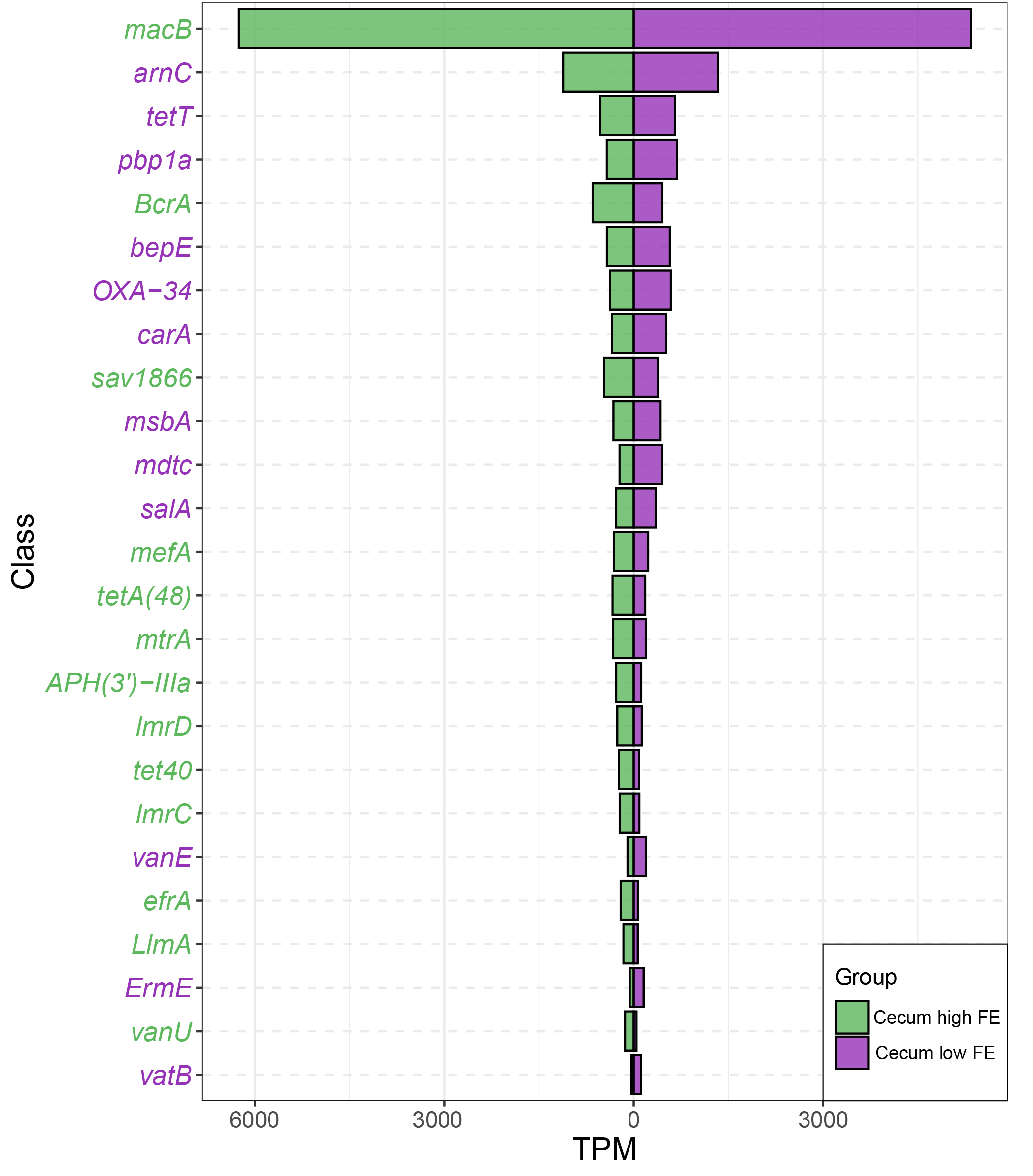

Supplement: FIGURE S13 — The abundance of ARG biomarkers of the high and low FE groups in cecum microbial community. [file Image_13.JPEG]

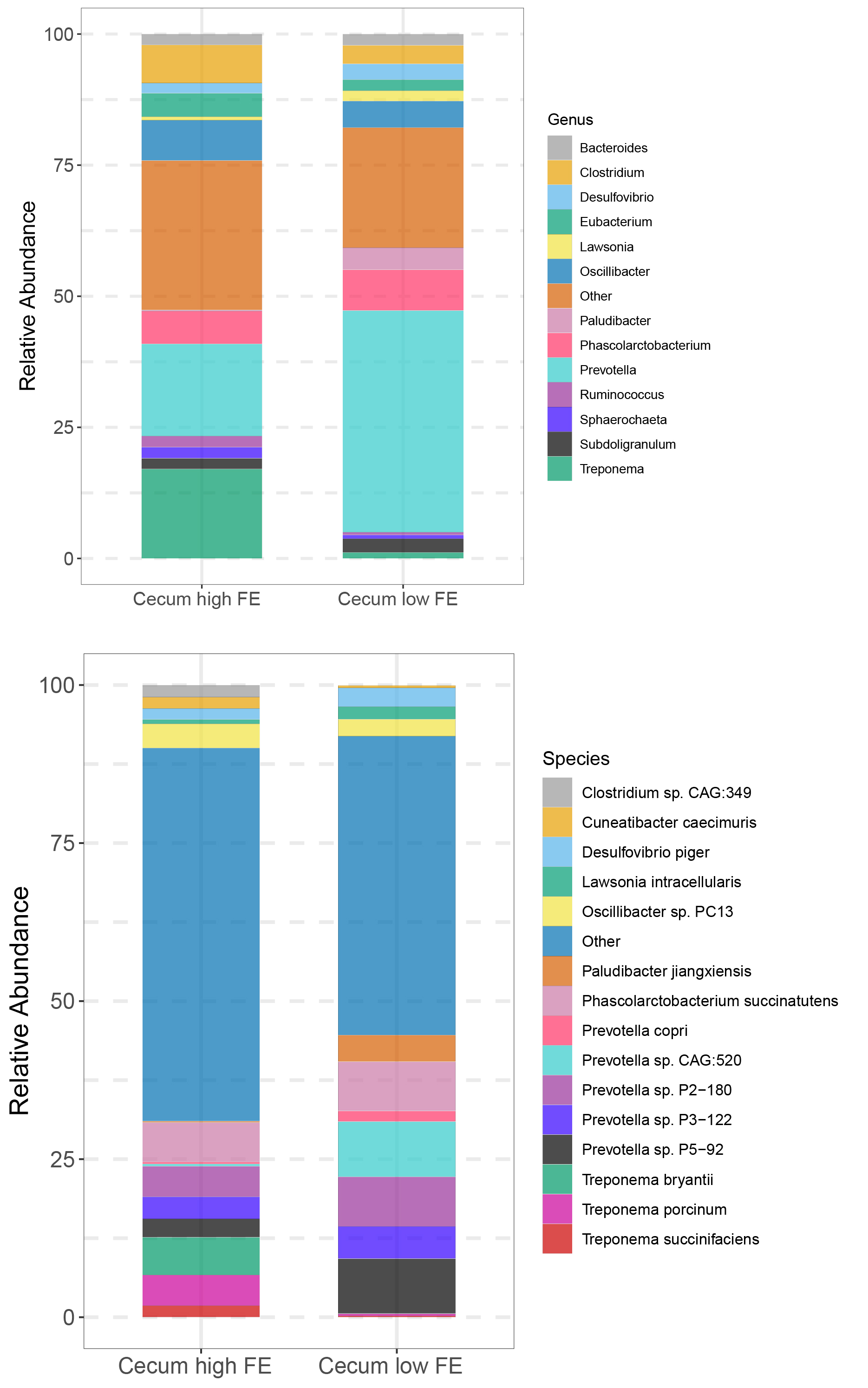

Supplement: FIGURE S14 — The relative abundance of top 10 abundant contributors of macB resistant gene between the high and low FE groups in cecum microbiota. [file Image_14.JPEG]
